# Molecular Study of Benzimidazole Resistance in *Teladorsagia circumcincta* Isolated from Sheep in North of Iran

**Published:** 2019

**Authors:** Rahim NEMATI, Aliasghar BAHARI, Pezhman MAHMOODI, Alireza SAZMAND

**Affiliations:** 1. Department of Pathobiology, Faculty of Veterinary Science, Bu-Ali Sina University, Hamedan, Iran; 2. Department of Clinical Sciences, Faculty of Veterinary Science, Bu-Ali Sina University, Hamedan, Iran

**Keywords:** Sheep, Anthelmintic resistance, *Teladorsagia*, Single nucleotide polymorphism, SNP, β-tubulin

## Abstract

**Background:**

Resistance to benzimidazole (BZ) compounds is common in *Teladorsagia circumcincta* populations in sheep and goats worldwide. Given the importance of anthelmintic resistance and shortage of information on single nucleotide polymorphisms (SNPs) in this prevalent nematode in Iran, this study was conducted.

**Methods:**

From June to September 2016, abomasa of 139 sheep of different sexes and ages in Amol City slaughterhouse, northern Iran were examined for isolation of nematodes. Totally 45 male *T. circumcincta* confirmed by both microscopical and nested-PCR-RFLP methods were included in this study. Susceptibility or resistance of each single *T. circumcincta* worm to benzimidazoles was assessed using allele-specific PCR.

**Results:**

Frequency of genotypes in the present study were 33.33% heterozygote BZ and 66.67% BZ homozygote sensitive. No homozygote resistant worm was found.

**Conclusion:**

Resistance against BZs in *T. circumcincta* of sheep has occurred at a low prevalence in the north of Iran. However, mutated genes might get dominant under drug selection in future. Hence, periodic investigations for early detection of mutated alleles in nematode populations using accurate and sensitive molecular methods such as PCR-RFLP is recommended.

## Introduction

Gastrointestinal parasitism is one of the most important causes of production losses in small ruminants (1). Although anthelmintic therapy used to prevent and control such infections, this may lead to the development of resistance in parasitic helminths which is becoming a serious problem for the livestock industry worldwide (2). In fact, development of resistance in nematodes of veterinary importance is influenced by a number of host-related physiological and environmental factors but importantly can result from operational factors such as frequent treatment with the same group of anthelmintics or single-drug regimens, anthelmintics-underdosing and mass treatment (3). Moreover, global warming has been also associated with development of resistance in certain nematodes (4).

The pathogenic abomasal parasite, *Teladorsagia circumcincta,* is common in domestic and wild sheep, goat, gazelle and camels in Iran (5–9). Frequent and non-principled use of anthelminthics that might reach five times a year has led to resistance of trichostrongylid nematodes to common broad-spectrum benizimidazole (BZs) and imidazothiazoles compounds (4, 10–12).

Other than morphological and physiological differences such as size and fertility in sensitive vs resistant *T. circumcincta* worms (13), genetic basis of resistance has attracted a lot of attention. BZ resistance in *T. circumcincta* has been associated with several independent single nucleotide polymorphisms (SNPs) in the isotype-1 β-tubulin gene at codons F167Y (14) and F200Y (15). However, resistance in other helminths such as *Haemonchus contortus* and *Trichuris trichiura* is correlated to one extra SNPs polymorphisms at codon E198A (16). Recently an additional polymorphism in substitution of glutamate for leucine (E198L) was found in *T. circumcincta* in Ireland albeit with a low frequency (17). This finding gives weight to the existence of additional determinants of resistance other than known mutations.

Benzimidazoles are used for more than 40 years as treatment or preventive chemotherapy of sheep nematodes in Iran. However, PCR-based studies performed on the status of BZ resistance in the nematode populations of sheep are limited to few reports on worms with unknown origin and/or unknown year of sampling (18–22).

Given the importance of anthelmintic resistance and shortage of information on SNP polymorphisms of *T. circumcincta*, the present study was conducted to evaluate benzimidazole resistance in this nematode isolated from sheep in north of Iran. Furthermore, a historical mini-review on the anthelmintic efficacy studies in Iran is presented in the discussion section and some key points for better planning of next studies are suggested.

## Materials and Methods

### Study area, isolation of worms and microscopic analysis

From June to September 2016, 139 sheep of both sexes (48 males and 91 females) and different ages (<6 months, *n*=7; 6 months–1 year, *n*=42; 1 year<, *n*=90) slaughtered at Amol abattoir, Mazandaran Province, north of Iran were randomly chosen. Abomasa of individual sheep were removed, put in plastic bags and transferred to the laboratory of Veterinary Office of Amol city under cold chain within hours. Recovered parasite specimens were collected in separate microtubes containing PBS and transferred to the Laboratory of Parasitology, Faculty of Veterinary Science, Bu-Ali Sina University of Hamedan, Iran. Adult male *T. circumcincta* worms were then specified according to morphological characters under light microscope (23) and stored in ethanol in separate microtubes at −20 °C until further analysis.

### Molecular identification of nematodes

DNA extraction from 45 isolated male *T. circumcincta* confirmed by light microscopy was carried out using a commercial DNA extraction kit (Yekta Tajhiz Azma, Iran) according to the manufacturer’s instruction. To confirm the identification of the nematodes, a previously described nested-PCR-RFLP method targeting isotype 1 *β*-tubulin gene was used (24). A negative control contained no DNA was used in all PCR runs. The nested-PCR products were digested using *Rsa*I restriction enzyme to confirm the identities of isolated nematodes (24).

### Allele-specific PCR

To determine the susceptibility or resistance of isolated *T. circumcincta* to benzimidazoles, a previously introduced allele-specific PCR was performed (25).

## Results

The results of the nested-PCR reactions and RFLP analysis confirmed the identity of all nematodes as *T. circumcincta*.

Allele-specific PCRs showed that the BZ resistance allele was present in 15 (33.33%) out of 45 examined *T. circumcincta*. No homozygous resistant (BZ^RR^) genotype was found. The other 30 nematodes were homozygous susceptible (BZ^SS^=66.67%). Table 1 shows resistance allele frequencies according to age and sex of the examined sheep.

**Table 1:** Frequency of *T. circumcincta* benzimidazole resistance alleles based on age and sex of sheep in the north of Iran

***Variable***	SS^*^	RS^**^	RR^***^	***Total***
Age < 1 year	11	4	-	15
Age > 1 year	19	11	-	30
Male	9	2	-	11
Female	21	13	-	34
**Total**	**30**	**15**	**-**	**45**

* Homozygous susceptible

** Heterozygous

*** Homozygous resistant

## Discussion

In the present study, resistance to benizimidazole compounds was examined in 45 *T. circumcincta* nematodes isolated from 139 sheep in north of Iran by the detection of alleles responsible for resistance.

The emergence of resistance against anthelmintic drugs is an important issue, several studies have been carried out in Iran. Anthelminthics efficacy examinations started as early as 1959 with testing lead arsenate in the treatment of ovine monieziosis (26). After that, efficacy of different compounds such as diethylcarbamazine citrate, emetine hydrochloride, thiabendazole, tetramisole, Lugol’s Iodin, ivermectin, levamisole and albendazole against nematodes in naturally infected ruminants in Iran was evaluated (10–12, 27–29). In a chain of field studies, resistance of *Teladorsagia*, *Trichostrongylus* and *Haemonchus* to levamisole and albendazole was examined in sheep of Khuzestan Province, southwestern Iran. These studies included sheep flocks from all parts of the province including mountainous, hilly and plain areas. Resistance of *T. circumcincta* and *T. vitrinus* to levamisole was found in 66.67% of studied flocks (10). Resistance of *T. circumcincta* and *Marshallagia marshalli* to albendazole in 27% of studied flocks was also observed (11). Methods of aforementioned works were copromicroscopical.

PCR-based detection of resistance alleles in sheep nematodes against commonly used benzimidazoles in Iran started in 2007. However, the researchers mainly focused on methodology other than field studies (21, 22), in some articles authors did not state year or area of sampling (18, 19, 21, 22) and in one study researchers pooled worms from each study area and proceeded the PCR-RFLP on extracted DNA from the pools (20). The relationship between treatment background and β-tubulin gene polymorphism of *H. contortus* was studied in seven regions of Khuzestan Province and researchers found that restriction profiles of β-tubulin gene in *H. contortus* varied in sheep with different treatment backgrounds (20). BZ resistance of adult *H. contortus* isolated from abomasa of sheep and goats was investigated in Khuzestan, Isfahan and Mazandaran provinces using a PCR-RFLP method which targeted point mutations at codons 167, 198 and 200 (18, 19). None of the tested specimens were found resistant.

In the present study, frequency of genotypes were 33.33% BZ ^RS^ and 66.67% BZ ^SS^ in *T. circumcincta* of north of Iran. Sensitive diagnostic approaches such as PCR-RFLP are crucial as if the BZ-resistant nematodes reach to medium level in natural population, no reversion is possible even if another anthelmintic agent without any cross-resistance is used (30). In the only previous PCR-based study on the BZ resistance in field isolates in Iran, adult *T. circumcincta* from 35 untreated and 40 albendazole-treated sheep were examined (22). BZ ^RR^ genotype was found in 5 worms (6.66%) collected from BZ-treated sheep. In the latter study, the year and area of isolation of worms were not stated in the article so comparison of the results of that work with ours is not possible. However, presence of mutated genes in BZ ^RR^ isolates even at a low frequency and heterozygote BZ ^RS^ isolates in this study and a previous study from Iran (31.11% and 82.66%) (22), can lead to dominancy under drug selection in future.

In this study, it was not possible to collect accurate information about history of anthelminthic therapy in the studied sheep brought to slaughterhouse. Although in a previous study one group of sheep was treated before, but the time interval of therapy-to-study was not stated clearly (22). Therefore, for future studies detailed information about treatment background of sample population might help in better interpretation of results.

In this study breeds and geographical origin of sheep that worms were isolated from were not recorded. Generally, the emergence rate of anthelminthic resistance varies geographically in accordance with climatic and environmental factors, parasite species and treatment regimens adopted in various regions (31). This is why fecal egg count reduction test (FECRT) is recommended for selecting the anthelmintic of choice in each region or even flock of livestock. Recently, polymorphism in DRB1 and DBR2 loci of the major histocompatibility complex, which plays a key role in immune responses, has been found to be associated with various gastrointestinal nematodes’ fecal egg count including *Teladorsagia* in Ghezel sheep breed in Iran (32, 33). Therefore, potential value of markers such as Ovar-DRB as indicator of parasite resistant sheep breeds should be taken into account in applied animal breeding programs. For future studies, studying correlations between developed resistance and breed of animals is suggested.

In Iran, *T. circumcincta* has been reported from various herbivorous species (6–9, 34). Cross-species transmission of gastrointestinal nematodes between domestic and wild animals occurs normally (35). Therefore, transmission of worms with resistance genes from/to susceptible livestock species is likely. Hence, mapping of resistant population of worms will be useful for quarantine legislation as animal movement is an important factor in distribution of drug resistant helminths (1).

Reports on resistance to major classes of anthelmintics such as the benzimidazoles, macrocyclic lactones and imidazothiazoles/tetrahydropyrimidines from all parts of the world are numerous. Discovery of a new class of anthelmintics, the amino-acetonitrile derivatives, in 2008 (36) gave a lot of hope but did not last long as there are growing evidence on failure of monepantel against *T. circumcincta* (37). However, advancement of diagnostic tools, development of vaccines, sustainable use of existing anthelminthics with application of tests like FAMACHA for selective therapy and improvement of control practices will help for better control of helminth infections by 2030 (38).

## Conclusion

Resistance against BZs in *T. circumcincta* of sheep has occurred at a low prevalence in the north of Iran. However, periodic investigations for early detection of mutated alleles in nematode populations using accurate and sensitive molecular methods such as PCR-RFLP will be of great importance to maintain the efficacy of currently available anthelmintics and prevent emergence of resistance in Iran. Furthermore, molecular-based investigations on resistance to other major classes of antihelminthics in Iran are suggested.

## References

[1] Roeber F Jex AR Gasser RB Impact of gastrointestinal parasitic nematodes of sheep, and the role of advanced molecular tools for exploring epidemiology and drug resistance-an Australian perspective. Parasit Vectors. 2013;6:153. 23711194 10.1186/1756-3305-6-153 PMC3679956

[2] Furtado LFV de Paiva Bello ACP1 Rabelo ÉML Benzimidazole resistance in helminths: from problem to diagnosis. Acta Trop. 2016;162:95–102. 27338184 10.1016/j.actatropica.2016.06.021

[3] Shalaby HA Anthelmintics resistance; how to overcome it? Iran J Parasitol. 2013;8(1):18–32. 23682256 PMC3655236

[4] Kaplan RM Vidyashankar AN An inconvenient truth: global worming and anthelmintic resistance. Vet Parasitol. 2012;186(1–2):70–8. 22154968 10.1016/j.vetpar.2011.11.048

[5] Eslami AH Nabavi L Species of gastrointestinal nematodes of sheep from Iran. Bull Soc Pathol Exot Filiales. 1976;69(1):92–5. 1036478

[6] Eslami A Meydani M Maleki S Zargarzadeh A Gastrointestinal nematodes of wild sheep (*Ovis orientalis*) from Iran. J Wildl Dis. 1979;15(2):263–5. 480517 10.7589/0090-3558-15.2.263

[7] Skerman KD Shahlapoor AA Eslami AH Eliazian M Observations on the incidence, epidemiology, control and economic importance of gastro-intestinal parasites of sheep and goats in Iran. Arch Razi Inst. 1970;22:187–96.

[8] Sazmand A Joachim A Parasitic diseases of camels in Iran (1931–2017)- A literature review. Parasite. 2017;24:21. 28617666 10.1051/parasite/2017024 PMC5479402

[9] Eslami A Rahbari S Nikbin S Gastrointestinal nematodes of gazelle, *Gazella subgutturosa*, in Iran. Vet Parasitol. 1980;7:75–8.

[10] Gholamian A Eslami A Nabavi L Rasekh AR A field survey on resistance of gastero-intestinal nematodes to levamisole in sheep in Khuzestan province of lran. J Fac Vet Med Uni Tehran. 2006;61(1):7–13. [In Persian with English summary].

[11] Gholamian A Eslami A Nabavi L Rasekh AR Galedari H A field survey on resistance to albendazole in gasterointestinal nematodes of sheep in Khozestan province of Iran. J Fac Vet Med Uni Tehran. 2007;62(1):45–51. [In Persian with English summary].

[12] Gholamian A Eslami A Nabavi L Rasekh AR Galedari H A survey on drug resistance in gastrointestinal nematodes of sheep using larval development assay. Iran Vet J. 2008;4(3):48–57.

[13] Leignel V Cabaret J Massive use of chemotherapy influences life traits of parasitic nematodes in domestic ruminants. Funct Ecol. 2001;15(5):569–74.

[14] Silvestre A Cabaret J Mutation in position 167 of isotype 1 β-tubulin gene of Trichostrongylid nematodes: role in benzimidazole resistance? Mol Biochem Parasitol. 2002;120(2):297–300. 11897135 10.1016/s0166-6851(01)00455-8

[15] Elard L Humbert JF Importance of the mutation of amino acid 200 of the isotype 1 β-tubulin gene in the benzimidazole resistance of the small-ruminant parasite *Teladorsagia circumcincta*. Parasitol Res. 1999;85(6):452–6. 10344538 10.1007/s004360050577

[16] Ghisi M Kaminsky R Mäser P Phenotyping and genotyping of *Haemonchus contortus* isolates reveals a new putative candidate mutation for benzimidazole resistance in nematodes. Vet Parasitol. 2007;144(3–4):313–20. 17101226 10.1016/j.vetpar.2006.10.003

[17] Keegan Jason D. Good Barbara de Waal Theo Genetic basis of benzimidazole resistance in *Teladorsagia circumcincta* in Ireland. Ir Vet J. 2017; 70: 8. 28228936 10.1186/s13620-017-0087-8 PMC5307811

[18] Nabavi R Shayan P Shokrani H Evaluation of benzimidazole resistance in *Haemonchus contortus* using comparative PCR-RFLP methods. Iran J Parasitol. 2011;6(2):45–53. 22347287 PMC3279878

[19] Shokrani H Shayan P Eslami A Nabavi R Benzimidazole-resistance in *Haemonchus contortus*: new PCR-RFLP method for the detection of point mutation at codon 167 of isotype 1 β-tubulin gene. Iran J Parasitol. 2012;7(4):41–8. 23323090 PMC3537475

[20] Gholamian A Galehdari H Eslami A Nabavi L Study of β-tubulin gene polymorphisms in *Haemonchus contortus* isolated from sheep populations in Khouzestan, southwestern Iran. Iran J Vet Res. 2007;8(3):239–43.

[21] Shayan P Borji H Eslami A Zakeri S Isolation of DNA from a single helminth using new developed kit in Iran and its PCR analysis. Iran J Parasitol. 2007;2(2):34–9.

[22] Shayan P Eslami A Borji H Innovative restriction site created PCR-RFLP for detection of benzimidazole resistance in *Teladorsagia circumcincta*. Parasitol Res. 2007;100(5):1063–8. 17136564 10.1007/s00436-006-0357-y

[23] Taylor MA Coop RL Wall RL Veterinary Parasitology. 4th ed Oxford; Ames, Iowa: Wiley Blackwell; 2016.

[24] Silvestre A Humbert JF A molecular tool for species identification and benzimidazole resistance diagnosis in larval communities of small ruminant parasites. Exp Parasitol. 2000;95(4):271–6. 11038310 10.1006/expr.2000.4542

[25] Glisić S Alavantić D A simple PCR method for rapidly detecting defined point mutations. Trends Genet. 1996;12(10):391–2. 8909130 10.1016/s0168-9525(96)90094-3

[26] Maghami G Alavi A Khalili K Monieziose des ovins en Iran et son traitement par l’arseniate de plomb. Arch Razi Inst. 1959;11(1):44–7.

[27] Shahlapour A Eslami A Eliazian H Comparative anthelmintic tests in sheep and goats infected with gastro-intestinal nematodes and lungworms in Iran. Trop Anim Health Prod. 1970;2(4):223–34.

[28] Eslami A Efficacy of ivermectin against *Setaria digitata* in cattle. J Vet Parasitol. 1998;12(1):58–9.

[29] Hosseini SH Eslami A Safari M Evaluation of antihelminte efficacy of ivermectin against gastrointestinal nematods with emphasis on *Bunostomum trigonocephalum* in naturally infected sheep. Fac Vet Med Uni Tehran. 2000;55(1):39–41. [In Persian with English summary].

[30] Leignel V Silvestre A Humbert JF Cabaret J Alternation of anthelmintic treatments: a molecular evaluation for benzimidazole resistance in nematodes. Vet Parasitol. 2010;172(1–2):80–8. 20570048 10.1016/j.vetpar.2010.04.023

[31] Falzon LC O’Neill TJ Menzies PI A systematic review and meta-analysis of factors associated with anthelmintic resistance in sheep. Prev Vet Med. 2014;117(2):388–402. 25059197 10.1016/j.prevetmed.2014.07.003

[32] Hosseinzadeh S Rafat S Moghaddam G Microsatellite polymorphism in DRB2 gene and its relation to *Haemonchus contortus* parasites fecal egg count in Iranian Ghezel sheep. Iran J Ruminant Health Res. 2016;1(1):31–9.

[33] Valilou RH Rafat SA Notter DR Fecal egg counts for gastrointestinal nematodes are associated with a polymorphism in the MHCDRB1 gene in the Iranian Ghezel sheep breed. Front Genet. 2015;6:105. 25852746 10.3389/fgene.2015.00105 PMC4371757

[34] Nabavi R Eslami A Shorkani HR Bokaei S Shayan P Saadati D Study on the prevalence, intensity, seasonal dynamics of abomasal helminths in sheep from different climatic zones of Iran. World Appl Sci J. 2011;12(4):441–5.

[35] Walker JG Morgan ER Generalists at the interface: nematode transmission between wild and domestic ungulates. Int J Parasitol Parasites Wildl. 2014;3:242–50. 25426420 10.1016/j.ijppaw.2014.08.001 PMC4241528

[36] Kaminsky R Ducray P Jung M A new class of anthelmintics effective against drug-resistant nematodes. Nature. 2008; 452(7184):176–80. 18337814 10.1038/nature06722

[37] Scott I Pomroy WE Kenyon PR Lack of efficacy of monepantel against *Teladorsagia circumcincta* and *Trichostrongylus colubriformis*. Vet Parasitol. 2013;198(1–2):166–71. 23953148 10.1016/j.vetpar.2013.07.037

[38] Vercruysse J Charlier J Van Dijk J Control of helminth ruminant infections by 2030. Parasitology. 2018;145(13):1655–64. 29415781 10.1017/S003118201700227X

